# The prevalence of depression, stress and anxiety symptoms in patients with chronic heart failure

**DOI:** 10.1186/s13033-021-00467-x

**Published:** 2021-05-12

**Authors:** Nqoba Tsabedze, Jamie-Leigh Hayes Kinsey, Dineo Mpanya, Vanessa Mogashoa, Eric Klug, Pravin Manga

**Affiliations:** Division of Cardiology, Department of Internal Medicine, School of Clinical Medicine, Faculty of Health Sciences, Charlotte Maxeke Johannesburg Academic Hospital, University of the Witwatersrand, 5 Jubilee Road, Parktown, Johannesburg, 2193 South Africa

**Keywords:** Depression, Stress, Anxiety, Chronic Heart Failure, Mental health, Psychosocial stressors

## Abstract

**Background:**

Mental health illnesses are associated with frequent hospitalisation and an increased risk of all-cause mortality. Despite the high prevalence of depression in patients with chronic heart failure (CHF), there is a paucity of data on this subject from low and middle-income countries (LMIC). The aim of this study was to determine the prevalence of depression, anxiety, and stress symptoms in patients attending a dedicated CHF clinic.

**Methods:**

A prospective study was conducted at an outpatient heart failure clinic in a tertiary academic centre. The study participants completed a Depression, Anxiety and Stress (DASS-21) questionnaire to screen for the presence and severity of depression, anxiety and stress symptoms. Furthermore, the Minnesota Living with Heart Failure Questionnaire (MLHFQ) was completed and used to evaluate the impact of CHF on health-related quality of life (QoL). Descriptive statistics were used to describe patients' characteristics and logistic regression analysis to identify predictors of symptoms of depression.

**Results:**

The study population comprised of 103 patients, predominantly female (62.1%) with a median age of 53 (interquartile range 38–61) years. Symptoms of depression were reported by 52.4%, with 11.6% reporting symptoms suggestive of extremely severe depression. Anxiety was diagnosed in 53.4% of patients and extremely severe anxiety was reported by 18.4% of patients. Fifty patients were classified as stressed, and only 7.7% had extremely severe stress. More than half of the patients (54.4%) were in New York Heart Association functional class I. The mean left ventricular ejection fraction in the entire cohort was 30% (SD =  ± 11.1%). In the multivariable logistic regression model, the MLHFQ score [odds ratio (OR) 1.04, 95% CI:1.02–1.06, p = 0.001] and the six-minute walk test [OR 0.99, 95% CI: 0.98–0.99, p = 0.014] were identified as independent predictors of depression.

**Conclusion:**

Depression and anxiety symptoms were found in over half of patients attending the CHF clinic. We recommend that mental health screening should be routinely performed in patients with CHF. Prospective, adequately powered, multicentre studies from LMIC investigating the impact of depression, anxiety and stress on CHF outcomes such as health-related QoL, hospitalisation and mortality are required.

## Introduction

Chronic heart failure (CHF) is a clinical syndrome associated with a significant reduction in the quality of life (QoL) of the patients affected [1]. Improving the health-related QoL is essential in managing patients with CHF, particularly since most patients value the QoL improvements more significantly than prolonging survival [2, 3]. Furthermore, mental health disorders may lead to an overall decreased health-related QoL due to non-compliance to medical therapy and follow-up clinic appointments. It has also been established that CHF accompanied by depression, anxiety, and stress is associated with an increased rate of heart failure-related hospitalisation and mortality [4–6].

Studies conducted in low and middle-income countries (LMIC) have demonstrated a positive correlation between mental health disorders and low levels of education, a poor socioeconomic status, and financial stress [7, 8]. According to studies done in other LMIC, we hypothesize that the prevalence of depression, anxiety, and stress in our patients with CHF is likely to be higher than that of patients residing in high income countries [9, 10]. This study aims to determine the prevalence of depression, anxiety, and stress symptoms in patients diagnosed with CHF in a tertiary academic hospital in Johannesburg, South Africa.

## Methods

We prospectively recruited consecutive patients attending the CHF outpatient clinic at the Charlotte Maxeke Johannesburg Academic Hospital (CMJAH), in Johannesburg, South Africa, between 01 January and 31 September 2016. The study commenced after receiving institutional ethical clearance and signed informed consent from all study participants. We included adults 18 years of age and older. The patients enrolled could all read and understand English. All study participants were provided with instructions on how to complete the English version of the Depression, Anxiety and Stress (DASS-21) questionnaire and the Minnesota Living with Heart Failure Questionnaire (MLHFQ). These questionnaires were completed while patients were awaiting their clinical consultation at the CHF clinic.

The DASS-21 questionnaire is a validated tool designed to measure the emotional states of depression, anxiety, and stress. Each scale contains seven items. The depression scale assesses dysphoria, hopelessness, devaluation of life, self-deprecation, lack of interest/involvement, anhedonia and inertia. The anxiety scale assesses autonomic arousal, skeletal muscle effects, situational anxiety and subjective experience of anxious affect; while the stress scale assesses difficulty in relaxation, nervous arousal, irritability and lack of patience. The final score is obtained by multiplying the total score in each of the three scales by two [11].

The MLHFQ evaluates the impact of heart failure on the patients’ health-related QoL. It is a disease-specific questionnaire comprising of 21 items rated on a six-point Likert Scale, representing different degrees of the impact of CHF on health-related QoL in the month prior to the current clinic visit, as perceived by the patient from 0 (none) to 5 (very much). The total score ranges between 0 and 105, where a higher score represents a poor QoL [12].

Sociodemographic factors (age, gender, ethnicity, employment status) and clinical parameters (blood pressure, body mass index, presence of heart failure symptoms) were collected using a data collection sheet designed for this study. Blood pressure measurements were taken from the right upper arm before and after the six-minute walk test. The weight and height measurements were also taken from each study participant. The rest of the clinical data was obtained from the CHF clinic outpatient file.

### Ethics approval

Research approval was granted by the Durban University of Technology (DUT) Research Ethics Committee as JH was a bachelor of technology student registered at DUT, and this study formed part of her research report. Permission to access patient records was obtained from the relevant hospital authorities.

### Statistical analysis

Categorical variables were expressed as numbers and percentages. All categorical variables were compared for the study outcome using the Chi-square test. Continuous variables with a normal distribution were expressed as mean and standard deviation (SD). The median and interquartile ranges (IQR) were used for continuous variables with a non-normal distribution. We compared normally distributed continuous variables using the Student t-test, and the Wilcoxon rank-sum (Mann–Whitney) test was used to compare medians for data with a non-normal distribution. Both univariate and multivariate logistic regression analyses were done to identify predictors for symptoms of depression. Confidence intervals were calculated at 95% interval levels, and differences were considered statistically significant at a p-value less than 0.05. All statistics were generated with STATA MP Version 13.0 (StataCorp. Texas).

## Results

The study population comprised of 103 patients with a median age of 53 (IQR: 38–61) years. Sixty-four patients (62.1%) were female and (71.8%) were black. Hypertension was the most prevalent comorbidity, seen in 37 (35.9%) participants. Only 13 (12.6%) patients had a history of ischaemic heart disease. Unemployment was reported by 67.9% of patients. Our patients were overweight with a mean body mass index (BMI) of 29 (SD =  ± 6.2 kg/m^2^). More than half of the patients (54.4%) were in New York Heart Association (NYHA) functional class I. The mean left ventricular ejection fraction (LVEF) was 30% (SD =  ± 11.1%).

Of the study participants, 98 (95.1%) were treated with beta-blockers, 94 (91.2%) were on angiotensin-converting enzyme (ACE) inhibitors or angiotensin receptor blockers (ARB), 89 (80.2%) were treated with diuretics, and 76 (73.7%) with mineralocorticoid receptor antagonists. The rest of the demographic and clinical data are reported in Table 1.

**Table 1:** Demographic and clinical characteristics of patients with chronic heart failure

Depression				
Variable	Total (n = 103)	No (n = 49)	Yes (n = 54)	p-value
Age, years	53 (38–61)	54 (38–62)	53 (38–61)	0.982
Females, n (%)	64 (62.1)	27 (55.1)	37 (68.5)	0.161
Ethnicity, n (%)				0.923
Black	74 (71.8)	37 (75.5)	37 (68.5)	
White	12 (11.6)	5 (10.2)	7 (12.9)	
Indian	11(10.7)	4 (8.2)	7 (12.9)	
Mixed Ancestry	4 (3.9)	2 (4.1)	2 (3.7)	
Other	2 (1.9)	1 (2.0)	1 (1.9)	
Employment status, n (%)				0.021
Unemployed	70 (67.9)	27 (55.1)	43 (79.6)	
Part-time	9 (8.7)	5 (10.2)	4 (7.4)	
Permanent	24 (23.3)	17 (34.7)	7 (12.9)	
Vital signs				
Systolic BP	124 (110–137)	124 (110–140)	123 (109–134)	0.498
Diastolic BP	73 (66–85)	73 (69–85)	75 (66–84)	0.871
MAP (mmHg)	92.1 (13.9)	92.8 (12.7)	91.5 (15.1)	0.643
Pulse (bpm)	72 (64–79)	71 (64–78)	72 (63–79)	0.731
MLHFQ score	16 (0–40)	5 (0–17)	28 (10–54)	0.000
BMI (kg/m^2^)	29 (6.2)	29.5 (4.7)	28.8 (7.4)	0.555
Six-minute walk test (metres)	350 (300–450)	375 (325–450)	350 (300–425)	0.008
LVEF (%)	30.5 (11.1)	30.5 (10.4)	30.4 (11.9)	0.494
NYHA class, n (%)				0.004
I	56 (54.4)	35 (71.4)	21 (38.9)	
II	33 (32.0)	10 (20.4)	23 (42.6)	
III	14 (13.6)	4 (8.2)	10 (18.5)	
Symptoms, n (%)				
Orthopnoea	11 (10.7)	2 (4.1)	9 (16.7)	0.039
Dyspnoea	16 (15.5)	5 (10.2)	11 (20.4)	0.155
PND	10 (9.7)	3 (6.1)	7 (12.9)	0.242
Fatigue	17 (16.5)	6 (12.2)	11 (20.4)	0.267

Dichotomous variables are represented as absolute numbers and percentages (%). Data showed as mean and standard deviation (SD) for continuous variables with a normal distribution and as a median and interquartile range (25–75th percentile) for continuous variables with a skewed distribution.

*BP* blood pressure, *BMI* body mass index, *MAP* mean arterial pressure, *MLHFQ* Minnesota Living with Heart Failure Questionnaire, *NYHA* New York Heart Association, *PND* paroxysmal nocturnal dyspnoea, *LVEF* left ventricular ejection fraction

**Fig. 1:**
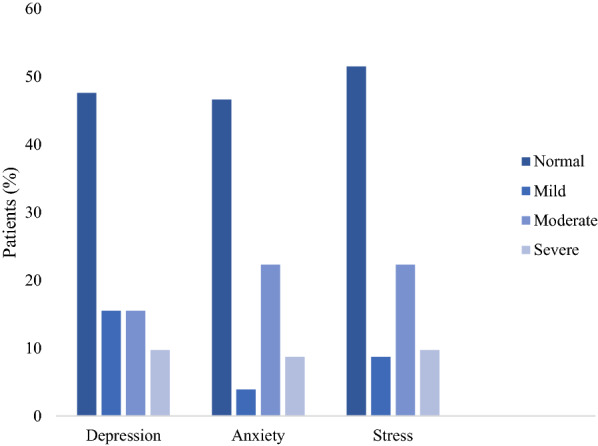
Graph showing chronic heart failure patients with depression, anxiety and stress stratified according to the severity

Fifty-four (52.4%) patients met the diagnostic criteria for depressive symptoms, with severe and extremely severe depressive symptoms reported in 10 (9.7%) and 12 (11.6%) patients, respectively (Fig. 1). Among the 54 patients with depressive symptoms, 72.7% also had symptoms of anxiety. Patients with symptoms of depression had a higher MLHFQ score [28 (10–54) vs. 5(0–17), p = 0.0004], and walked a shorter distance during the six-minute walk test [350 (300–425) vs. 375 (325–450) metres, p = 0.008]. The univariate logistic regression model demonstrated a positive linear relationship between symptoms of depression and the NYHA functional class II and III, a higher MLHFQ score, and orthopnea. In the multivariate logistic regression model, the MLHFQ score [odds ratio (OR) 1.04, 95% CI: 1.02–1.06, p = 0.001] and the six-minute walk test [OR 0.99, 95% CI: 0.98–0.99, p = 0.014] were identified as independent predictors of depression. (Table 2).

**Table 2:** Logistic regression analysis for predictors of depression

	Univariate regression			Multivariate regression		
	OR	p-value	95% CI	OR	p-value	95% CI
NYHA class II	3.83	0.004	1.53–9.60			
NYHA class III	4.17	0.029	1.16–14.97			
Permanent employment	0.26	0.008	0.09–0.71			
Six-minute walk test	0.99	0.005	0.98–0.99	0.99	0.014	0.98–0.99
MLHFQ score	1.04	0.000	1.02–1.06	1.04	0.001	1.02–1.06
Orthopnoea	4.50	0.054	0.97–20.83			

*CI* confidence interval, *MLHFQ* Minnesota living with heart failure questionnaire, *NYHA* New York Heart Association, *OR* odds Ratio

## Discussion

Depression is linked to the development and progression of CHF and other cardiovascular diseases (CVD) [13–16]. However, anxiety and stress have not been as clearly associated with poor clinical outcomes in CHF patients [17]. Despite the adverse impact of depression, anxiety, and stress on CHF patients, these disorders remain underdiagnosed and undertreated in this high-risk population [17, 18]. This problem is significant in patients residing in LMIC, who generally have less sophisticated health care systems, which are often unable to optimally screen, diagnose and manage CHF patients with these comorbidities [19].

In our study, 52.4% of patients had symptoms of depression. This is significantly higher compared to most studies in high-income countries. In a community-based study conducted in the United States of America, the prevalence of depression in CHF patients was 17% [20]. Furthermore, Haworth et al. studied 100 outpatients with heart failure in the United Kingdom and found the prevalence rate for anxiety and depression to be 18% and 29%, respectively [10]. The higher prevalence of symptoms of depression in our patients is likely influenced by socioeconomic factors unique to populations in LMIC. This is supported by two studies conducted in Ethiopia and Pakistan, where the prevalence of depression was reported in 51.1% and 60% of the participants, respectively [19, 21].

Depression and anxiety are common yet underreported in CHF and should not be overlooked when managing these patients. This is particularly important since both conditions have been associated with poor outcomes with more robust evidence for depression [18]. In a systematic review evaluating the prevalence, variance, and measurement tools for anxiety in patients with heart failure, the pooled prevalence from 38 studies ranged from 6.3 to 72.3% [5]. In our study, anxiety and depression coexisted in 72.7% of patients. Such a finding emphasizes the need to screen for both conditions since the treatment plan differs between the two conditions.

The use of screening tools, such as the 2 and 9-item Patient Health Questionnaires [22], improves the recognition of these psychiatric disorders in CHF patients, and this strategy is currently endorsed by the American and European heart failure societies [23, 24]. However, to accurately diagnose depression, anxiety and stress in CHF patients may be challenging due to the overlap of somatic symptoms shared between CHF and these psychiatric disorders. Hence, the use of formal diagnostic criteria such as the Diagnostic and Statistical Manual of Mental Disorders (DSM) and a structured clinical interview are still considered to be the best practice for the evaluation process [17, 18]. Furthermore, screening for psychiatric disorders in the acute admission or early post-discharge phase may help identify CHF patients with symptoms of depression, stress or anxiety. However, these symptoms may regress in the sub-acute and long-term period and may not represent a true psychiatric disorder [25]. It may also be an anticipated response for newly diagnosed CHF patients to present with these somatic symptoms.

Potential mechanisms linking depression and anxiety with poor heart failure outcomes include inflammation, autonomic dysfunction, enhanced platelet aggregation, endothelial dysfunction, poor diet, smoking, and reduced physical activity [17]. There is a paucity of data on the prevalence of stress in CHF patients. However, there is a well-established relationship between prolonged emotional or physical stress and activation of the autonomic nervous system, increasing the likelihood of myocardial infarction, arrhythmias, heart failure and sudden cardiac death [26, 27].

In a study by Gottlieb et al., involving 155 patients with heart failure, 48% of the participants met the diagnostic criteria for depression, based on the Beck Depression Inventory, and had a mean MLHFQ score of 54 (SD =  ± 24) [28]. In our study, patients with symptoms of depression also had a higher median MLHFQ score of 28 (IQR: 10–54) (p = 0.0004). Moreover, in the multivariate logistic regression model, a higher MLHFQ score was an independent predictor of depression (p = 0.001).

The six-minute walk test is a simple, reproducible test sensitive to changes in functional capacity [29]. Furthermore, a self-perceived feeling of depression is a determinant of a shorter six-minute walk test [30]. In our study, a longer distance walked during the six-minute walk test was associated with a reduced likelihood of symptoms of depression (p = 0.014).

Other published predictors of depression in heart failure include advanced age, the female gender, a low socioeconomic status, a previous depressive episode, smoking, a higher NYHA functional class and unmarried status [20, 21, 31–33]. In our cohort, the NYHA functional class was not an independent predictor of depression. Thirty-nine percent of patients in NYHA functional class I had symptoms of depression, compared to only 18.5% in NYHA functional class III, suggesting that other confounding factors, such as the socioeconomic status, could play an important role in acquiring symptoms of depression.

This study highlights the high prevalence of depression, anxiety and stress in CHF outpatients from a LMIC. In our cohort, a higher MLHFQ score and a short six-minute walk test distance were independent predictors of depressive symptoms. The high prevalence of depression, anxiety and stress in CHF patients warrants routine clinical screening during follow-up visits and the collaborative management of these patients by psychologists, psychiatrists, and cardiologists.

## Study limitations

This study was limited by a small sample size and patient enrolment from a single medical centre. Patients were recruited over a short period, and only included study participants who could communicate in English. In our study, the DASS-21 questionnaire was the only tool used to assess symptoms of depression, stress and anxiety and patients were not subsequently interviewed by a psychologist or psychiatrist. We are mindful that the prevalence of the symptoms of depression is significantly influenced by the measurement tool used. However, there is no data suggesting that the DASS-21 questionnaire under or overestimates the prevalence of depression, stress, and anxiety. Lastly, this study was a cross-sectional analysis with no patient follow-up data to assess for progression of symptoms of depression, anxiety, or stress. Despite these limitations, our study demonstrates that depression, anxiety and stress symptoms are common in CHF patients residing in a LMIC.

## Conclusion

Symptoms of depression and anxiety were found in more than 50% of patients with CHF. Based on these findings, mental health screening should be considered in CHF patients, with appropriate referral pathways to psychologists and psychiatrists. We recommend that prospective, multicentre studies from LMIC be conducted using multiple tools to screen for depression, anxiety, and stress and investigate their impact on CHF outcomes.
